# Validation of the Indonesian version of multiple sclerosis quality of life-54 (MSQOL-54 INA) questionnaire

**DOI:** 10.1186/s12955-019-1190-1

**Published:** 2019-07-12

**Authors:** Riwanti Estiasari, Syarly Melani, A. A. A. Agung Kusumawardhani, David Pangeran, Yuhyi Fajrina, Kartika Maharani, Darma Imran

**Affiliations:** 1 Department of Neurology, Faculty of Medicine Universitas Indonesia, Jl. Salemba Raya no. 6, Jakarta, 10430 Indonesia; 2 Dr. Cipto Mangunkusumo General Hospital, Jakarta, Indonesia; 3 Department of Psychiatry, Faculty of Medicine Universitas Indonesia, Jakarta, Indonesia

**Keywords:** Multiple sclerosis, Quality of life, MSQOL, Validation

## Abstract

**Background:**

Quality of life assessment of patients with multiple sclerosis (MS) is not routinely performed in Indonesia due to the unavailability of the validated Indonesian version of a specific instrument. The objective of this study was to transculturally adapt and validate the Indonesian version of the MSQOL-54 (MSQOL-54 INA) questionnaire.

**Methods:**

The transcultural adaptation was conducted by performing a standardized forward-backward method. Psychometric analysis was performed by assessing the reliability (Cronbach α), internal validation (item internal consistency and item discriminant validity), and external validation by measuring the correlation with a clinical factor such as EDSS and other demographic factors.

**Results:**

Reliability test with Cronbach α showed good internal consistency (> 0.7) at each component, except for health perception (0.665) and social function (0.433). Construct validity using computation of correlation coefficient showed internal consistency in accordance with the original MSQOL-54 standard dimension, except for energy and role limitation due to emotional problems components. External validation with EDSS showed negative correlation on almost all components, except for sexual function, but both composite scores were statistically significant.

**Conclusion:**

MSQOL-54 INA questionnaire has good internal reliability and is proven to be valid and well-accepted by Indonesian MS patients. Therefore, it can be used by Indonesian clinicians for more comprehensive MS management.

**Electronic supplementary material:**

The online version of this article (10.1186/s12955-019-1190-1) contains supplementary material, which is available to authorized users.

## Background

Multiple sclerosis (MS) is a progressive neurodegenerative autoimmune disorder affecting the myelin sheath of the central nervous system [1]. In 2013, The Multiple Sclerosis International Federation (MSIF) reported that its prevalence had increased from 2.1 million in 2008 to 2.3 million in 2013 [2]. In Indonesia, while this disease is still considered rare, its number begins to rise gradually in these recent years.

MS is predominantly diagnosed in women of productive age, in which various long-term physical and psychosocial disabilities are inevitable and have potential to impact the productivity as well as the abilities in performing activities of daily living (ADL) [3, 4]. Previous studies have proved that MS patients had a lower quality of life compared with others due to their limitation and disabilities in performing ADL [5, 6].

Quality of life (QOL) is an individual perception on their position in life following the cultural system and moral values in their living environment, which is associated with each’s vision, standards, expectations, and attention [7]. The role of health-related QOL (HRQOL) assessment is to plan the next clinical management, to measure the outcome in the clinical study, to assess the health needs in a population, and as a source for budget allocation for health problems [8, 9]. Quality of life is a relevant outcome to be evaluated in patients with chronic diseases, such as multiple sclerosis [10].

In the previous years, various instruments have been developed for measuring QOL in MS patients, such as Multiple Sclerosis Quality of Life-54 (MSQOL-54), Hamburg Quality of Life Questionnaire in Multiple Sclerosis (HAQUAMS), Functional Assessment of Multiple Sclerosis (FAMS), Multiple Sclerosis Quality of Life Inventory (MSQLI),the Multiple Sclerosis International Quality of Life (MUSIQOL) and the Multiple Sclerosis Impact Scale (MSIS) [9, 11]. Only MSQOL-54 and MSQLI consists of generic HRQOL instruments and several specific questions in accordance with the clinical manifestations and targets of MS management.

One of the most commonly used instruments worldwide is the MSQOL-54 questionnaire [12, 13]. This instrument was initially developed in the United States by Vickrey et al. [14] and has been transcultural adapted ever since. Up to date, this instrument has been available in numerous languages, such as Italian, Turkish, French Canadian, Bosnian, Serbian, and Slovenian, in the effort to overcome the language barrier in implementing the instrument [15–20]. The MSQOL-54 is a structured, self-reported questionnaire which can generally be filled by the patient with little or no assistance. Overall, this instrument has good internal consistency and test-retest reliability. The outcome of this questionnaire reflects the physical and mental health condition in the form of physical and mental health composite score, respectively [13–16, 18].

In Indonesia, the assessment for QOL in MS has not routinely been performed due to the unavailability of Indonesian version questionnaire. This study aimed to produce the Indonesian version of MSQOL-54 (MSQOL-54 INA) that was valid and reliable to be implemented in Indonesian scientific community for clinical practice and research. Besides, this questionnaire was also expected to be used for the evaluation of MS progression in areas with inadequate diagnostic facilities. The objectives of this study were to translate the MSQOL-54 into Indonesian and assess its validity and reliability in Indonesian MS patients.

## Methods

This was a cross-sectional study with the study population of all MS patients visiting dr. Cipto Mangunkusumo General Hospital, Jakarta. All MS patients attending the hospital during June–September 2018 and fulfilling the study criteria were included using consecutive non-random sampling method.

### Inclusion and exclusion criteria

Inclusion criteria were (1) MS patients fulfilling the 2010 McDonalds diagnosis criteria, (2) age > 18 years old, able to read and write in Indonesian; (3) providing written consent to be recruited into the study. Exclusion criteria were subjects undergoing relapse in the previous month or having psychiatric disorders or other chronic diseases (diabetes mellitus, hypertension, etc.).

### Instrument

The QOL in this study was assessed using MSQOL-54 questionnaire consisting of fifty-four questions. This questionnaire comprised SF-36, the instrument for general QOL assessment, and the additional eighteen specific questions for MS patients. The examination result contained two domains of composite scores, reflecting the physical health (PH) and mental health (MH), independent to each other. The separation of domains and components were adapted from the original MSQOL-54 version proposed by Vickrey et al. [14].

### Translation and cultural adaptation process of MSQOL-54 INA

Due to the availability of the validated Indonesian version of the SF-36 questionnaire, translation and transcultural adaptation processes were only performed on eighteen specific questions for MS patients [7, 21]. Standardized forward-backward translation method was performed for the production of the MSQOL-54 INA. The steps of the translation process were (1) forward translation of the original MSQOL-54 questionnaire to Indonesian by two groups of professional translators blinded to each other; (2) reconciliation of the translated questionnaire by a panel team, consisting of an Indonesian neurologist and psychiatrist expert in their field, to compose conceptually and semantically similar questionnaire with the original version; (3) back-translation of reconciled questionnaire to English by two different translators blinded to each other; (4) reconciliation of the translated result to produce the targeted version. The panel team then created agreement regarding the culture of the source and targeted instrument following four aspects, which is semantic, idiomatic, daily experience, and conceptual. This step produced a questionnaire which was then tested to MS patients (expert committee); (5) the reconciled questionnaire was then tested to 10 MS subjects consecutively to gain inputs from the subjects’ perception regarding the grammatical clarity, easiness to be understood, and any suggested alternate words during the test. (6) composing the last version of the questionnaire [17, 22, 23].

### Collection of the data

The samples meeting the inclusion criteria were asked to fill the MSQOL-54 INA questionnaire directly attended by medical staff as necessary. During the process, the time needed to complete the questionnaire was documented as well as whether the patient needed help in reading and writing the answers were documented. Simultaneously, Expanded Disability Status Scale (EDSS) score was measured to assess the patient’s disability level.

### Psychometric analysis

Psychometric analysis for MSQOL-54 INA questionnaire was performed, consisting of reliability, internal validity, and external validity tests. Reliability of internal consistency was measured by using the Cronbach alpha coefficient (α) with a range of 0–1. The higher the result, the better the reliability. An instrument was defined to have consistent internal reliability if α was ≥0.7 [7, 24]. The Cronbach alpha value from the original research by Vickrey et al. was also used as a comparison.

Internal validity was assessed with content validity and construct validity. Content validity had been performed during the transcultural adaptation process by the neurologist and the psychiatrist. Construct validity was performed by executing computation of correlation coefficients, which was by measuring the correlation coefficients of each domain and its original domain (item internal consistency (IIC)) compared with the opposing domain (item discriminant validity (IDV). The IIC of > 0,4 and higher than the IDV proved the construct validity. In addition, the principal component analysis using direct Oblimin method was performed to assess whether the separation of domains in the original study by Vickrey et al. suited the condition in Indonesia.

Kaiser-Meyer-Olkin (KMO) and Bartlett sphericity test were performed beforehand. The principal component analysis was analyzed if the KMO score 0.6 and higher and the Bartlett sphericity test showed *p* < 0.05.

External validity was assessed by measuring the association of each component and the composite scores with EDSS score. The score of each dimension and domain of MSQOL-54 was expected to be negatively correlated with EDSS. Pearson (r) and Spearman (p) correlation coefficients were used for measuring the association of composite scores with the clinical and demographic variables [7].

## Results

This study involved 43 MS patients in which female subjects (79,1%) were predominant. All other characteristics of the subjects are displayed in Table 1.

**Table 1:** Characteristics of Indonesian MS patients for the validation of MSQOL-54 INA questionnaire (*n* = 43)

Characteristics	n (%)
Gender	
• Male	9 (20.9%)
• Female	34 (79.1%)
Education	
• < 12 years	14 (32.6%)
• > 12 years	29 (67.4%)
Occupation	
• Unemployed/housewife	20 (46.5%)
• Student	6 (14.0%)
• Employee	12 (27.9%)
• Professional	5 (11.6%)
Status	
• Married	16 (37.2%)
• Single	24 (55.8%)
• Widow/widower	3 (7.0%)
	Median (Min-Max)
Age (years)	30 (20–61)
Duration (years)	4.0 (0.2–13.0)
Relapses in the recent one year	1.0 (0.0–3.0)
EDSS score	3.0 (0.0–8.0)

*EDSS* expanded disability status scale, *MS* multiple sclerosis, *MSQOL-54 INA* Multiple Sclerosis Quality of Life-54 Indonesia version

Before administering the MSQOL-54 INA questionnaire, the questionnaire had already passed the content validity phase (Table 2). All subjects could comprehend the questions and do not meet any significant language barrier in general during the testing. The average time to complete the questionnaire was 14.4 ± 6.34 min. Of all subjects, 76.7% could fill the questionnaire independently. Assistance was mainly necessary for subjects with visual and upper extremities impairment. Missing data were found only on the questions regarding sexual function and satisfaction with sexual function since these questions were only filled by married subjects (*n* = 17).

**Table 2:** Summary of problems in content validity process

No. of question	Original Question	Problems	Solution
44	Have you had trouble with your memory	Back translated as “do you have difficulties in remembering”	Modification of translation from “mengingat (verb)” to “daya ingat (noun)”
42–45	Choices “a good bit of the time” and “some of the time”	In Indonesia, the meaning of both words were very similar with “sometimes”	In accordance with the adapted Indonesian version of SF-36, those two choices were merged and the weight for all choices were readjusted.

The mean and Cronbach alpha values are shown in Table 3. The lowest mean value was found in the role limitation due to physical problems component (37.21 ± 22.82) while the highest mean value was found in the sexual function component (67.65 ± 39.52). The mean of PH composite score was 51.84 ± 15.05 whereas the mean of the MH composite score was 53.36 ± 15.57. In general, the Cronbach alpha value from this study was above 0.70 except for health perception (0.66) and social function component (0.43).

**Table 3:** Statistical description and results of reliability analysis of MSQOL-54 INA

Component	Number of questions	Mean ± SD	Cronbach alpha	
			This study	Vickrey et al.[14]
Physical function	10	60.00 ± 29.78	0.92	0.96
Health perception	5	50.70 ± 17.95	0.66	0.80
Energy	5	57.67 ± 18.11	0.72	0.84
Role limitation-physical	4	37.21 ± 22.82	0.91	0.86
Pain	3	65.50 ± 25.04	0.88	0.92
Sexual function	4	67.65 ± 39.52	0.97	0.85
Social function	3	62.79 ± 20.92	0.43	0.75
Health distress	4	43.26 ± 18.58	0.84	0.91
Overall QOL	2	65.70 ± 18.04	0.82	0.86
Emotional well-being	5	62.91 ± 22.05	0.87	0.87
Role limitation-emotional	3	42.95 ± 21.15	0.84	0.84
Cognitive function	4	46.22 ± 22.91	0.81	0.90
Change in health	1	63.37 ± 29.55	*	*
Satisfaction with sexual function	1	54.41 ± 30.92	*	*
PH composite score	8 components	51.84 ± 15.05	0.78	
MH composite score	5 components	53.36 ± 15.57	0.79	

*Cronbach Alpha could not be computed because the scale is based one single item

*MSQOL-54* Multiple Sclerosis Quality of Life-54, *QOL* quality of life, *PH* Physical health, *MH* Mental Health

The results of internal validity assessment showed that all components have item internal consistency of > 0.40. Calculation of item discriminant validity showed that energy component, which was a part of PH composite, also showed high values at MH domain whereas role limitation due to emotional problems component showed higher values at PH composite (Additional file 1: Table S1).

In addition, principal component analysis using direct Oblimin rotations was conducted twice. The first analysis was performed with the exclusion of change in health and satisfactory with sexual function components (*N* = 17), which was in conjunction with the original study by Vickrey, et al. [14] The second analysis was performed with the additional exclusion of sexual function due to the less response from the subjects compared with the other components (Additional file 2: Table S2). The first analysis had KMO value of 0.47 whereas second analysis produced KMO value of 0.81. From the second factor analysis, there were discrepancies compared with the original domain proposed by Vickrey, et al. in which role limitation due to emotional problem was moved to the PH domain whereas health perception, energy, and social function were more suitable to the MH domain.

Correlation with EDSS score (Table 4) showed statistically significant results only at physical functioning, pain, role limitation due to physical problems, role limitation due to emotional problems, PH composite score, and MH composite score components.

**Table 4:** External validity: Correlation between MSQOL-54 INA questionnaire and EDSS

Component	Pearson correlation	p
Physical function	− 0.85	< 0.001
Health Perception	− 0.25	0.1
Energy	− 0.12	0.44
Role limitation-physical	−0.52	< 0.001
Pain	−0.36	0.02
Sexual function	0.19	0.45
Social function	−0.2	0.19
Health distress	−0.17	0.28
Overall QOL	−0.19	0.21
Emotional well-being	−0.09	0.53
Role limitation-emotional	−0.49	0.001
Cognitive function	−0.14	0.36
Change in health	−0.17	0.26
Satisfaction with sexual function	−0.11	0.67
PH composite score	−0.54	< 0.001
MH composite score	−0.3	0.05

*EDSS* expanded disability status scale, *MSQOL-54* Multiple Sclerosis Quality of Life-54, *QOL* quality of life

Correlation between composite scores and other clinical and demographic factors, such as age, duration of disease, and number of relapses in the recent year showed no significant correlation (Table 5).

**Table 5:** External validity: Correlation between MSQOL-54 questionnaire and clinical and demographic factors

Component	Age		Duration of disease		Number of relapse	
	r	p	r	p	r	p
Physical function	−0.12	0.45	−0.37	0.02	−0.08	0.59
Health Perception	−0.05	0.74	0.001	0.95	−0.11	0.48
Energy	0.16	0.31	−0.07	0.65	−0.09	0.56
Role limitation-physical	0.12	0.44	−0.12	0.43	−0.05	0.77
Pain	−0.15	0.34	−0.09	0.57	0.003	0.98
Sexual function	−0.68	0.003	−0.23	0.37	0.23	0.37
Social function	−0.005	0.97	−0.14	0.38	−0.09	0.56
Health distress	0.06	0.69	−0.06	0.69	−0.05	0.77
Overall QOL	−0.02	0.88	−0.08	0.59	−0.11	0.49
Emotional well-being	0.11	0.43	−0.04	0.81	−0.05	0.73
Role limitation-emotional	0.09	0.55	−0.22	0.16	−0.01	0.93
Cognitive function	0.05	0.73	−0.03	0.84	−0.12	0.45
Change in health	−0.17	0.26	0.14	0.37	−0.36	0.02
Satisfaction with sexual function	−0.37	0.14	−0.04	0.88	0.16	0.53
PH composite score	0.07	0.65	−0.21	0.18	−0.01	0.93
MH composite score	−0.04	0.78	−0.20	0.19	−0.04	0.77

*MSQOL-54* Multiple Sclerosis Quality of Life-54, *QOL* quality of life, *PH* Physical Health, *MH* Mental Health

## Discussion

### Main findings of the study

Almost all patients could complete the questionnaire within the recommended range of 11–18 min [14], which described that the questions were easy to understand and manageable. The missing data in this study could be minimized as little as possible with the presence of clinicians who could help the subjects as needed during the test. However, missing data were found in sexual function and satisfaction with sexual function components since only those who were married were willing to answer those components. This condition was closely related to local cultural perception which believed that sexual matters were taboo, especially for unmarried individuals. For the married individuals, the questions regarding sexual were distressing, too private or sensitive to be answered. This statement was in conjunction with the good result of sexual function and satisfaction with sexual function components in this study.

In general, this study found high reliability value, which showed that this questionnaire was internally consistent. The low Cronbach α scores on health perception and social function components could result from the less number of questions assessing those components; for example, the social function component consists of only three questions.

From the internal validity assessment, satisfactory results were obtained. Observing the internal validity using principal component analysis, there were several discrepancies between the domain analysis and the proposed domain by Vickrey, et al. These findings could still not be explained in this study, but a larger sample was still needed to observe the consistencies of these findings as these two components were those having the least reliability score.

Assessing the external validation using EDSS scores, the negative correlation was found at almost all components, except for sexual function and satisfaction with sexual function components. This might be due to the perception of MS patients’ population in Indonesia who perceived questions regarding sexual problems as taboo, leading to potentially unreported issues. Physical health-related questions were more statistically significant in correlation with EDSS since EDSS was the most common clinical assessment used for the physical disability assessment of MS patients [5, 25]. Similar results were also shown by the previous study by Brunet et al. which found that the disability assessed with EDSS only had the correlation with physical function domain at RAND-36 questionnaire [26].

### Comparison with other similar studies

In accordance with the studies by Idiman E et al. in Turkish [16], Catic T et al. in Bosnia and Herzegovina [18], Pekmezovic T et al. in Serbian [19], Stern B et al. in Slovenian [20], and Solari A et al. in Italian [15], the questions regarding sexual function and satisfaction with sexual function were the most potential missing data. This problem arose due to the feeling of embarrassment in admitting the issues regarding sexual, which is in accordance with the respective culture. However, different from the other studies, this study could not collect many subjects to fill this part due to the small MS population in Indonesia, most of which were still unmarried.

Compared to the original study conducted by Vickrey et al. in the United States, there were similarities that health perception and social function domain had the lowest Cronbach α [14]. However, the value in this study was less than 0.7, which was similar with the studies conducted in Serbia, Slovenian, Israel, and Iran [5, 19, 20, 27]. Stern et al. explained that the low Cronbach α value at health perception component might be caused by the nature of the questions which included wide and unspecific health evaluation aspects which led to relatively lower consistency [20].

Regarding the analysis of IIC and IDV, this study found that energy and role limitation due to emotional problems component were more suitable to show MH domain rather than the PH domain. These results were also reported by previous psychometric studies [5, 20, 27]. This described that in those population, as well as in Indonesian MS population, low energy score was more perceived as mental problems whereas role limitation due to emotional problems was more perceived as physical problems in conjunction with role limitation due to physical problems.

In view of the analysis of principal component analysis, there was only a study focusing on this analysis. In this study by Stern B, et al. in Slovenian [20], the discrepancies between domains and components were only observed in energy and role limitation due to emotional problems. In contrast, besides those two components, the component of health perception and social function was also assigned into different domain compared with the original domain proposed by Vickrey et al. [14]. This result was not found on a similar study by Stern B et al. The small sample size and the lower reliability number may explain this discrepancy, but further research was needed.

The study of Solari A et al. in Italia, Idiman E, et al. in Turkish, and Catic C, et al. in Bosnia supported the correlation of EDSS and PH composite score in this study [15, 16, 18]. These studies also found that EDSS was negatively correlated with the PH composite score, which supported the fact that EDSS described the health in physical aspects.

In this study, the correlation between composite scores and other clinical and demographic factors, such as age, duration of disease, and the number of relapses in the past one year did not show significant correlation. Similar results were found in a study in Turkey in which there was no significant correlation between composite scores and age, gender, education level, marital status, and health insurance [16].

### Limitations and strengths of the study

This study provided a readily-used of MSQOL-54 INA which can be implemented for the Indonesian population. The study results also proved that this instrument was valid and reliable, which can be trusted by potential users. In addition, this study also provided psychometric properties of MSQOL-54 INA questionnaire in Indonesia MS population. However, this study had a small sample size, which may impact on the reliability score for some components. This limitation was inevitable because this disease was still rare even though the study was conducted in the national referral hospital in Indonesia. Additionally, confirmatory factor analysis was not applied in this study. Similar studies with more subjects in the future were needed to prove the consistency of this study result.

Another limitation of this study was associated with the content validity process, in which an independent third party did not supervise the translation process as proposed by Acquardro C et al. [17].

### Implications of the study results for clinical management of MS patients

This is the first study focusing on the specific QOL instrument for MS patients. This instrument can generally be used for Indonesian MS patients with good validity and reliability. Besides, this validated questionnaire may become one of the tools to evaluate the success of the therapy as well as the baseline for the clinician to focus not only on the patients’ clinical aspects but also on the medical rehabilitation, cognition, and psychiatric issues.

### Suggestions on future research in the field

This study had produced an adapted MSQOL-54 INA questionnaire with well-considered content validity and high reliability in the aspect of internal consistency on most components. Further studies with larger sample size are needed to prove the internal consistency of some components, particularly health perception and social function that had lower Cronbach alpha value and sexual function that had a smaller sample size. In addition, this instrument can also be used as a measure for assessing the success of therapy, which has not been analyzed in this study. Further additional analysis with confirmatory analysis method was also recommended to be performed with larger sample size.

## Conclusion

The Indonesian version of the MSQOL-54 questionnaire is easy to be completed and well-accepted by Indonesian MS patients. Psychometric analysis showed entirely satisfactory results. Those support the use of this questionnaire for quality of life assessment in relation with MS patients’ health in Indonesia. This questionnaire is expected to be able to help clinicians to manage MS patients comprehensively.

## Additional files

Additional file 1: **Table S1.** Results of item internal consistency and item discriminant validity of MSQOL-54 INA questionnaire. (DOCX 16 kb)

Additional file 2: **Table S2.** Principal factor analysis of MSQOL-54 components. (DOCX 17 kb)

## Data Availability

The datasets used and/or analysed during the current study are available. from the corresponding author on reasonable request.

## Abbreviations

ADL: Activities of daily living
EDSS: Expanded Disability Status Scale
HAQUAMS: Hamburg Quality of Life Questionnaire in Multiple Sclerosis
HRQOL: Health-related Quality of Life
IDV: Item discriminant validity
IIC: Item internal consistency
KMO: Kaiser-Meyer-Olkin
MH: Mental Health
MS: Multiple sclerosis
MSIS: Multiple Sclerosis Impact Scale
MSQOL-54: Multiple Sclerosis Quality of Life-54
MUSIQOL: Multiple Sclerosis International Quality of Life
PH: Physical Health
QOL: Quality of life
