# Supplementary material for: Validation of the Indonesian version of multiple sclerosis quality of life-54 (MSQOL-54 INA) questionnaire
Source: Health Qual Life Outcomes. 2019 Jul 12;17:120. doi: 10.1186/s12955-019-1190-1 (PMC6626390; doi:10.1186/s12955-019-1190-1)
Supplement: Supplementary file 1 — Table S1. Results of item internal consistency and item discriminant validity of MSQOL-54 INA questionnaire. (DOCX 16 kb) [file 12955_2019_1190_MOESM1_ESM.docx]

Additional file 1: Table S1. Results of item internal consistency and item discriminant validity of MSQOL-54 INA questionnaire

| Component | Respective domain | Opposing domain | item internal consistency | item discriminant validity |
| --- | --- | --- | --- | --- |
| Physical function | PH | MH | 0.79 | 0.58 |
| Health Perception | PH | MH | 0.78 | 0.74 |
| Energy | PH | MH | 0.75 | 0.84 |
| Role limitation-physical | PH | MH | 0.77 | 0.68 |
| Pain | PH | MH | 0.63 | 0.46 |
| Sexual function | PH | MH | 0.47 | 0.31 |
| Social function | PH | MH | 0.61 | 0.55 |
| Overall QOL | MH | PH | 0.68 | 0.64 |
| Emotional well-being | MH | PH | 0.86 | 0.71 |
| Role limitation-emotional | MH | PH | 0.73 | 0.75 |
| Cognitive function | MH | PH | 0.62 | 0.36 |
| Health distress | PH and MH | PH and MH | 0.65 | 0.78 |

*Item internal consistency was calculated by comparing each component to its respective domain whereas item discriminant validity was calculated by comparing each component to the opposing domain.*

*MSQOL-54: Multiple Sclerosis Quality of Life-54; QOL: quality of life; PH: Physical Health; MH: Mental Health*
