# Supplementary material for: Validation of the Indonesian version of multiple sclerosis quality of life-54 (MSQOL-54 INA) questionnaire
Source: Health Qual Life Outcomes. 2019 Jul 12;17:120. doi: 10.1186/s12955-019-1190-1 (PMC6626390; doi:10.1186/s12955-019-1190-1)
Supplement: Supplementary file 2 — Table S2. Principal factor analysis of MSQOL-54 components. (DOCX 17 kb) [file 12955_2019_1190_MOESM2_ESM.docx]

Additional file 2: Table S2. Principal factor analysis of MSQOL-54 components

| Original domain by Vickrey, et al [14] | | Factor analysis^A^ (n=17) | | | Factor analysis^B^ (n=43) | | |
| --- | --- | --- | --- | --- | --- | --- | --- |
|  |  | PH | MH | C | PH | MH | C |
| KMO Sample Adequacy | | 0.47 | | | 0.812 | | |
| Bartlett’s Test of Sphericity | | p = 0.003 | | | p <0.005 | | |
| Physical function | PH | -0.59 | 0.16 | 0.43 | 0.92 | -0.08 | 0.78 |
| Health Perception | PH | -0.17 | 0.71 | 0.61 | 0.24 | 0.67 | 0.69 |
| Energy | PH | -0.39 | 0.58 | 0.65 | 0.06 | 0.87 | 0.82 |
| Role limitation-physical | PH | -0.89 | -0.08 | 0.75 | 0.88 | 0.02 | 0.78 |
| Pain | PH | -0.41 | 0.44 | 0.48 | 0.54 | 0.17 | 0.43 |
| Sexual function | PH | 0.17 | 0.56 | 0.28 | - | - | - |
| Social function | PH | -0.72 | -0.16 | 0.47 | 0.15 | 0.6 | 0.49 |
| Overall QOL | MH | 0.17 | 0.87 | 0.69 | 0.12 | 0.67 | 0.55 |
| Emotional well-being | MH | -0.17 | 0.83 | 0.81 | -0.16 | 0.99 | 0.84 |
| Role limitation-emotional | MH | -0.84 | 0.04 | 0.72 | 0.86 | 0.05 | 0.78 |
| Cognitive function | MH | -0.57 | 0.29 | 0.52 | 0.04 | 0.47 | 0.24 |
| Health distress | PH & MH | -0.14 | 0.6 | 0.43 | -0.12 | 0.87 | 0.65 |

*A: Analyzed with exclusion of* *change in health and satisfactory with sexual function components*.

B: *Analyzed with exclusion of* *change in health, satisfactory with sexual function and sexual function components*

*MSQOL-54: Multiple Sclerosis Quality of Life-54; QOL: quality of life; PH: Physical Health; MH: Mental Health; C: Communalities; KMO:* *Kaiser-Meyer-Olkin*
